# Effects of two different peptides on pentylenetetrazole-induced seizures in larval zebrafish

**DOI:** 10.1371/journal.pone.0308581

**Published:** 2025-04-25

**Authors:** Jhonathan Angel Araujo Fernández, Thatiane Cristina de Moura, Sabela Fernández Vila, Juan Andrés Rubiolo Gaytán, Iñaki López-Díaz, Soraya Learte-Aymamí, M. Eugenio Vázquez, Maria D. Mayán, Laura Sánchez, Claudia Vianna Maurer-Morelli

**Affiliations:** 1 Departamento de Genética Médica e Medicina Genômica, Faculdade de Ciências Médicas, Universidade Estadual de Campinas, UNICAMP, Campinas, Brazil; 2 Brazilian Institute of Neuroscience and Neurotechnology, Campinas, Brazil; 3 Departamento de Zoología Genética y Antropología Física, Facultad de Veterinaria, Universidad de Santiago de Compostela, Campus de Lugo, Lugo, Spain; 4 Centro Singular de Investigación en Química Biolóxica e,Materiais Moleculares (CiQUS), Departamento de Química Orgánica, Universidade de Santiago de Compostela, Santiago de Compostela, Spain; 5 CELLCOM Research Group, Nanomaterials and Biomedical Research Center (CINBIO) and Institute of Biomedical Research of Ourense-Pontevedra-Vigo (IBI), University of Vigo and Servizo Galego de Saúde (SERGAS). Edificio Olimpia Valencia, Campus Universitario Lagoas Marcosende, Pontevedra, Spain; ¤ Before: Instituto de Investigación Biomédica de A Coruña (INIBIC), Universidade da Coruña (UDC), A Coruña, Spain; University of Modena and Reggio Emilia, ITALY

## Abstract

Epilepsy is a common and severe neurological disease characterized by spontaneous and recurrent seizures. Although anti-seizure treatments are effective for most patients, approximately 30% remain pharmacoresistant. Moreover, uncontrolled seizures are associated with increased health risks and shortened life expectancy in individuals with refractory epilepsy. Preclinical studies play a crucial role in drug discovery, and the zebrafish (*Danio rerio*) have been successfully employed for this purpose. In this study, we utilized the zebrafish PTZ-induced seizure model to evaluate the effects of two peptides on seizure responses: Tripeptide (p-BTX-I) and the CX2 (a Cx43derivated peptide). Zebrafish larvae at 6 days post-fertilization were pre-treated with these peptides at various concentrations, depending on their experimental groups, 24h prior to seizure induction. We assessed seizure frequency, quantified swimming activity, measured transcript levels of genes related to inflammation and apoptosis (*il1b, tnfa, cox1, cox2a, il6, casp3a, casp9, baxa, bcl2a,* and *c-fos*), and analyzed the biodistribution of both peptides. Our results indicate that the Tripeptide exhibited anti-inflammatory and anti-apoptotic effects, particularly through reducing the expression of *il1b* and *casp9*. CX2 pre-treatment significantly downregulated inflammatory markers (*il1b, il6, tnfa,* and *cox1*). Biodistribution analysis confirmed that the CX2 peptide reached the zebrafish brain, suggesting a direct role in modulating seizure-related pathways. Our findings demonstrate that Tripeptide and CX2 peptides can modulate gene expression and mitigate molecular response associated with epileptic seizures in the zebrafish brain. These peptides thus represent promising candidates for future research aimed at developing novel anti-epileptic therapies. However, additional studies are required to evaluate their long-term efficacy, elucidate underlying mechanisms of action, and explore potential translational applications.

## Introduction

Epilepsies are a common neurological disease characterized by recurrent unprovoked seizures, affecting approximately 70 million people worldwide [1,2]. Currently, anti-seizure drugs are effective for 66% of people with epilepsy in developed countries; however, more than 30% of patients are considered pharmacoresistant to conventional therapies [3–4]. Furthermore, uncontrolled seizures are associated with multiple risks, including injury, neuropsychological impairments, and reduced lifespan [5]. Consequently, identifying novel therapeutic agents capable of improving seizure management is of utmost urgency.

Pre-clinical trials play a pivotal role in evaluating the therapeutic efficacy and potential toxicological effects of new compounds using *in vivo, in vitro*, or *ex-vivo* approaches. The scalability and rapid generation of results significantly enhance the value of these studies. Within this context, zebrafish (*Danio rerio*) have emerged as an a key model organism, offering distinct advantages over other animal models in pre-clinical research [6–7]. These advantages include small size, low maintenance cost, high fecundity, and optical transparency during embryogenesis [8]. Furthermore, the zebrafish genome shares approximately 70% homology with the human genome, with 84% of known genes associated with human diseases such as epilepsy [9,10]. Zebrafish also align with the principles of the 3Rs philosophy (reduction, refinement, and replacement), particularly when studies are conducted below 5 days post fertilization (dpf) [11]. Today, zebrafish is widely recognized as a suitable model organism for studying human diseases, supported by validated behavioral repertoires and diverse phenotypic assessment methodologies [12–15].

In the context of epilepsy/seizure research, zebrafish represent a well-characterized seizure model, utilized in both adult and larval stages, including models created through genetic manipulation [13,16–19]. In 2005, Baraban and colleagues described a zebrafish larval model of seizure induced by the proconvulsant agent pentylenetetrazol (PTZ) [19]. Upon exposure of larvae at seven dpf to 15mM PTZ, the authors observed characteristic seizure behaviors, increased expression of the *c-fos* gene expression in the optic tectum, and electrographic seizure discharges. Notably, these seizure responses were attenuated by common anti-seizing drugs [19]. Thus, zebrafish have been increasingly employed as a valuable model organism for the discovery of novel natural or synthetic anti-seizure medications, as well as for drug repurposing, thereby significantly contributing to new insights into seizure-modulation mechanisms [20–22].

Given the advantages of zebrafish as a phenotypic-based drug screening model and recognizing the urgent need for novel therapeutic agents for seizure management, this study aimed to evaluate the effects of two distinct peptides on PTZ-induced seizures.

## Materials and methods

### Zebrafish maintenance and embryo acquisition

Wild-type adult zebrafish were obtained from the Zebrafish Laboratory and Husbandry at the School of Medical Sciences, Unicamp. The animals were housed in thanks of 30–50 liters at a density of two animals per liter of water. Thanks were maintained under controlled physicochemical conditions, including temperature (26±2°C), pH (7–7.5), ammonia levels (< 0.1 ppm), nitrite levels (< 0.2 ppm), and dissolved oxygen (4–11 ppm). A photoperiod cycle of 14 hours of light and 10 hours of darkness was maintained. Adult fish were fed three times daily with commercial flake food (Tetramin, Tetra, Blacksburg, VA, USA) and once daily with brine shrimp and paramecium. Embryos were collected following natural spawning and maintained in Petri dishes containing aquarium water under conditions of temperature and photoperiod identical to those of the adults. Larvae were fed paramecium starting from the fifth dpf. Ethical approvals were obtained from the Ethics Committee for Animal Research of the State University of Campinas (CEUA 4895–1/2019 and CEUA 5757–1/2021).

### Peptides

#### Tripeptide (p-BTX-I).

The peptide sequence (Glu-Val-Trp) was obtained from AminoTech – Research and Development (São Paulo, Brazil). Subsequently, the peptide was diluted in Milli-Q water (Merck KGaA, Darmstadt, Germany), aliquoted, and stored at -20°C. The employed concentrations (as listed in Table 1) were determined based on research conducted in cell models [23–24].The concentrations employed in the experiments (listed in Table 1) were determined based on previous research conducted in cell models [23,24].

**Table 1:** Peptides and their respective concentrations evaluated for their effects in the PTZ-induced seizure model.

Compound	Concentration
Tripeptide (p-BTX-I)	25, 10 and 5 µg/mL
CX2	0.5, 0.1 and 0.05 µM

#### CX2.

The CX2 (ARG-Cx43p) sequence corresponds to the C-terminal domain of the zebrafish Cx43 gene (PCT/EP2020/071242), granted by Dr. Maria D. Mayán. CX2 was assembled following standard Fmoc/tBu solid-phase MW-assisted peptide synthesis protocols [25–26], purified by reverse-phase HPLC, and their identity was confirmed by HPLC-MS(ESI). The concentrations used (as listed in Table 1) were determined through studies involving zebrafish models for regeneration and senescence. Like Tripeptide, CX2 was diluted in Milli-Q water, aliquoted, and stored at -20°C.

#### Peptides pretreatment.

At 6 dpf, zebrafish larvae were randomly placed into Petri dishes containing 25 larvae each. Larvae were then incubated for 24 hours in their respective peptide solutions and concentrations (as detailed in Table 1) before seizure induction. Temperature and photoperiod conditions matched those previously described for adult zebrafish. The experimental groups consisted of the Control Group (CG), PTZ Group (PTZ), Treatment 1, Treatment 2, and Treatment 3. Treatments 1–3 employed the previously determined concentrations mentioned above.

### Seizure induction by pentylenetetrazol

At 7 dpf, larvae were carefully transferred into a 96-well plate containing 100 µL of aquarium water, with one larva per well. Subsequently, 100 µL of 30 mM pentylenetetrazol (PTZ Sigma-Aldrich, St. Louis, MO, USA) was added to each well, resulting in a final concentration of 15 mM of PTZ. Larvae were exposed to PTZ for 20 minutes. Control group larvae underwent the same procedure but received PTZ-free water.

### Behavioral assay

Seizure behavior was assessed by visually counting seizures and quantitatively analyzing swimming behavior (total distance traveled and velocity) using EthoVision XT tracking software (Noldus, Wageningen, The Netherlands) during the 10-minute PTZ exposure. Visual observations were performed by an experienced observer who evaluated each larva twice to determine the number of seizures. The average of the two measurements per larva was used for statistical analysis. A seizure was considered complete only when the larva reached stage 3 seizure-like activity, characterized by a loss of posture, which served as the gold standard for classification according to criteria established by Baraban et al. (2005) [19].

### RNA extraction

Immediately following PTZ exposure, larvae were quickly cryoanesthetized and their heads were removed, collected, and incubated in TRIzol® (Invitrogen, Carlsbad, CA, USA). The biological material was lysed using the TissueLyzer equipment (QIAGEN, GmbH, Germany) at 25 beats per second (bps) for 2 minutes. Subsequently, samples were stored at -80 °C until further processing. Each experimental group consisted of five samples (n = 5), each sample was composed by pooling five larval heads to ensure sufficient biological material for RNA extraction. Total RNA extraction was performed using TRIzol® according to the manufacturer’s protocol. RNA concentration and quality were determined using an Epoch™ spectrophotometer (BioTek, Winooski, VT, USA) and gel electrophoresis.

### RT-qPCR

cDNA synthesis was performed using the High-Capacity cDNA Reverse Transcription kit (Applied Biosystems™, Califórnia, USA) following the manufacturer’s instructions. Quantitative PCR (qPCR) was carried out using the SYBR ® Green Master Mix reagent (Bio-Rad) using the ABI 7500 system (Applied Biosystems, Foster City, CA, USA). Primers for target genes (as listed in Table 2) were designed using the Primer–BLAST online tool (https://www.ncbi.nlm.nih.gov/tools/primer-blast/) from NCBI (National Center for Biotechnology Information) zebrafish database. qPCR reactions were conducted in triplicate, and gene expression was normalized against the housekeeping gene *eef1a1l1* [27]. Genes analyzed included markers from the inflammation pathway (*il1b, tnfa, cox1, cox2a* and *il6*), the apoptosis pathway (*casp3a, casp9, baxa,* and *bcl2a,* and neuronal activity (*c-fos*). The inflammation and apoptosis pathways are closely linked to the underlying mechanisms of seizure generation and progression. Therefore, we selected representative genes from these pathways for analysis. A total of five samples (n = 5) per group were analyzed, with each sample representing a pool of five larval heads. Data analysis was performed using 7500 software v2.03 (Applied Biosystems), and relative gene expression was calculated using the Livak and Schmittgen method (RQ = 2^-ΔΔCT) [28].

**Table 2:** Primer sequences designed using the Primer-Blast online tool.

Gene	Seq.Ref.NCBI	Forward	Reverse	Size (pb)
*eef1a1l1*	NM_131263.1	AGCAGCAGCTGAGGAGTGAT	CCGCATTTGTAGATCAGATGG	140
*il1b*	NM_212844.2	GCTGGAGATCCAAACGGATA	ATTTGACGGACTCGAAGGTG	85
*tnfa*	NM_212859.2	TCGGGTGTATGGAGGGTGTT	TTGATTGCCCTGGGTCTTATGG	96
*cox1*	NM_153656.2	CTGGGAGGCTTATTCCAACA	CCAGAAGTTTAGGGTCTGGAAG	119
*cox2a*	NM_153657.1	ACCAGGGCGTGTGTTTATCC	GTGAGAAGCTCAGGGGTAGTG	100
*il6*	NM_001261449.1	GGCATTTGAAGGGGTCAGGA	GCGTTAGACATCTTTCCGTGC	92
*casp3a*	NM_131877.3	CAGCTTGAACTACCCCAACA	AAGCTTTCGAAACACGTTCA	133
*casp9*	NM_001007404.2	GCGACAAGCTGGAGAAAAGA	GATGACCACACAGCAGTCGTA	140
*baxa*	NM_131562.2	GAGCTGCACTTCTCAACAACTTT	GAAGATCTCACGGGCCACTC	245
*bcl2a*	NM_001030253.2	ACTACCTGAACGGGCCACT	AAAACGGGTGGAACACAGAG	105
*c-fos*	XP_009291581.1	CGCAGCTCAATCCTACAACC	TCTTGTTTCGTTCACGACGTA	245

### Statistical analysis

Statistical analysis was conducted using GraphPad Prism version 5.0 (GraphPad Software, San Diego, CA, USA). Data were analysed using one-way ANOVA followed by the Bonferroni post hoc test for multiple comparisons. Results are presented as mean ± SEM. Differences were considered statistically significant at *p*<0.05.

### Bio-distribution

Bio-distribution analysis was performed using Tripeptide and CX2 labeled with tetramethylrhodamine-TMR and carboxytetramethylrhodamine – TAMRA, respectively. Larvae at 6 dpf were individually transferred to wells in a 96-well plate containing 100 µL of aquarium water. Next, 100 µL of either Tripeptide (20 µg/mL) or CX2 (1 µM) was added according to the experimental group, resulting in a final solution volume of 200 µL per well and final concentrations of 10 µg/mL for Tripeptide and 0.5 µM for CX2. Exposure duration for both peptide groups were 1, 6, 18, and 24 hours (n=3 each time point). Control larvae were similarly manipulated but were exposed only to aquarium water. During the experiment, the incubation temperature was maintained at 26±2°C. Following exposure, larvae were rapidly and carefully transferred to a Becker containing 0.02% tricaine (Tricaine, Sigma) for approximately 2 minutes. Before imaging, larvae were gently washed to remove residual skin fluorescence. Subsequently, larvae were placed in a Petri dish containing 1% agarose as a base along with a drop of water. Images were captured using a Multizoom AZ100 microscope (Nikon, Tokyo, Japan) at 2X magnification. Two images of each larva were obtained: one in black-and-white, and another capturing fluorescence emission only. Images were merged using NIS-Elements software (Nikon, Tokyo, Japan). All experimental procedures and protocols were approved by the Animal Care and Use Committee of Universidad de Santiago de Compostela and complied with Spanish standard guidelines (CEEA-LU-003) and Directive 2012/63/EU.

## Results

### Behavioral assay

**Fig 1:**
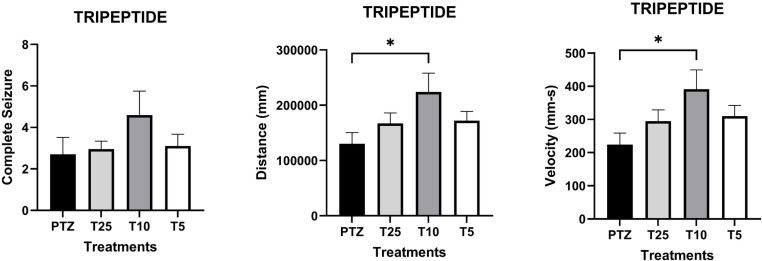
Effects of the Tripeptide treatment before pentylenetetrazol (PTZ)-induced seizures on seizure-like behaviors and swimming activity. Animals were exposed to the Tripeptide for 24 hours before to Pentylenetetrazol (PTZ)- induced seizures. Groups included: PTZ control (n=10); Tripeptide treatment at 25 µg/mL (T25; n=10); 10 µg/ml (T10; n=10); 5 µg/ml (T5; n=10). Animals were video-recorded during a 10-minute exposure to PTZ (15mM). **(A)** The number of seizures was scored by visual analysis twice per larva, with loss of posture serving as the criterion for a complete seizure event; **(B)** Distance traveled and **(C)** velocity for each larva were quantified using EthoVision XT locomotion tracking software (Noldus, Wageningen, The Netherlands). Data are presented as mean ± SEM. Statistical analyses were performed using one-way ANOVA, followed by the Bonferroni correction for multiple comparisons. Differences were considered significant at *p* < 0.05.

**Fig 2:**
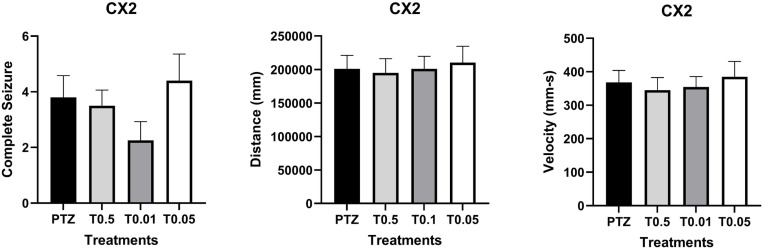
Effects of CX2 treatment before pentylenetetrazol (PTZ)-induced seizures on the number of seizure-like behaviors and swimming activity. Animals exposed to CX2 for 24 hours before Pentylenetetrazol (PTZ)- induced seizures. Groups included: PTZ control (n=10); CX2 treatment at 0.5 µM (T0.5; n = 9), 0.1 µM (T0.1; n = 10), and 0.05 µM (T0.05; n = 10). Animals were video-recorded during a 10-minute exposure to PTZ (15 mM). **(A)** The number of seizures was scored visually twice per larva, with loss of posture as the criterion for a complete seizure event. **(B)** Distance traveled and (C) velocity of each larva were quantified using EthoVision XT locomotion tracking software (Noldus, Wageningen, The Netherlands). Data are expressed as mean ± SEM. Statistical analyses were conducted using one-way ANOVA followed by Bonferroni correction for multiple comparisons. Differences were considered significant at *p* < 0.05.

Behavioral analysis revealed increased swimming activity in larvae treated with the tripeptide at 10 µg/mL compared to the PTZ group (Fig 1B and 1C). In contrast, treatment with CX2 did not produce statistically significant differences in swimming activity or seizure behavior compared to the PTZ group (Fig 2).

### Molecular assay

**Fig 3:**
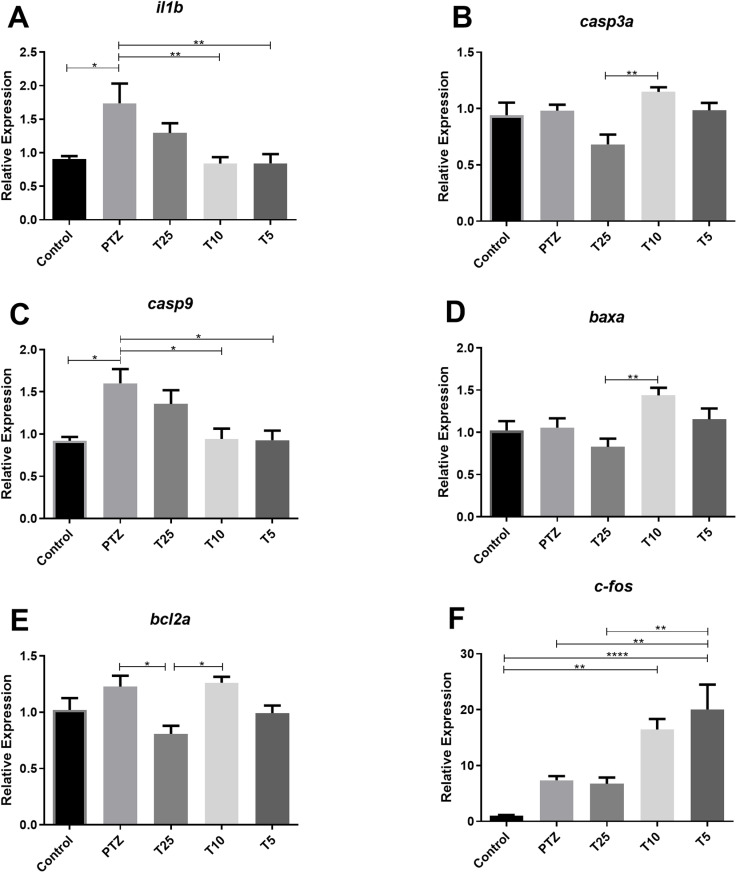
Expression levels of *il1b*, *casp3a*, *casp9*, *baxa*, *bcl2a,* and *c-fos* genes in zebrafish brain after pentylenetetrazol (PTZ)-induced seizures. Each treatment group was pre-exposed to the Tripeptide for 24 h, followed by 15 mM PTZ exposure for 20 min. Control and PTZ groups underwent identical handling procedures but were exposed to water (n = 5 per group). Data are presented as mean ± SEM. Statistical analyses were performed with the one-way ANOVA, followed by Bonferroni’s test for multiple comparisons. Differences were considered significant at p<0.05. Significance is indicated as follows: *p≤0.05; **p≤0.01; ****p≤0.0001. Groups: Control (Control), PTZ (pentylenetetrazol group), T25 (25 µg/mL treatment), T10 (10 µg/mL treatment), T5 (5 µg/mL treatment).

Molecular assays indicated the tripeptide downregulation of the expression of the *il1b* and *casp9* genes (Fig 3A and 3C), with significant effects observed at lower concentrations (10 µg/mL and 5 µg/mL). Additionally, at 10 µg/mL, transcript levels of the *casp3a*, *baxa,* and *bcl2a* genes were significantly elevated compared to certain treatment groups, though no significant difference was observed relative to the PTZ group (Fig 3B, D, and E). In contrast, *bcl2a* expression was significantly reduced at a concentration of 25 µg/mL compared to the PTZ group (p<0.05; Fig 3E). Furthermore, *c-fos* transcript levels increased at lower doses, with a significant upregulation observed at 5 µg/mL, compared to other groups (Fig 3).

**Fig 4:**
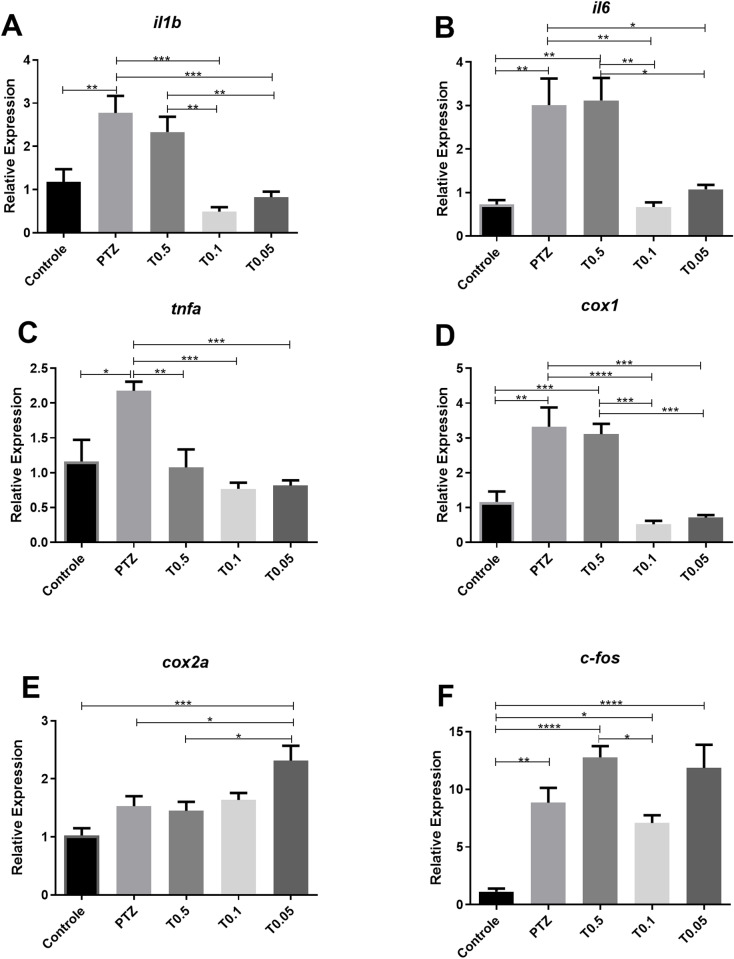
Expression levels of *il1b*, *il6*, *tnfa*, *cox1*, *cox2a* and *c-fos* genes in zebrafish brain after pentylenetetrazol (PTZ)-induced seizures. Each treatment group was pre-exposed to CX2 for 24 h, followed by exposure to 15 mM PTZ for 20 min. Control and PTZ groups underwent identical procedures but were exposed to water only (n = 5 per group). Data are presented as mean ± SEM. Statistical analyses were performed with the one-way ANOVA, followed by Bonferroni’s multiple comparisons test. Differences were significant at p<0.05. Significance indicators: *p≤0.05; **p≤0.01; ***p≤0.001; ****p≤0.0001. Groups: Control (Control), PTZ (pentylenetetrazol group), T0.5 (0.5 µM treatment), T0.1 (0.1 µM treatment), T0.05 (0.05 µM treatment).

Regarding CX2, a downregulation of the *il1b, cox1, il16, and tnfa* genes was observed at most tested concentrations compared to the PTZ group (Fig 4), with the most significant downregulation occurring at 0.1 µM. Conversely, the *cox2a* gene exhibited increased expression across all tested concentrations, reaching peak significance at 0.05 µM (p≤0.001). Additionally, *c-fos* transcript levels were elevated in the treatment groups compared to the control group, but did not differ significantly from the PTZ group (Fig. 4).

### Bio-distribution

**Fig 5:**
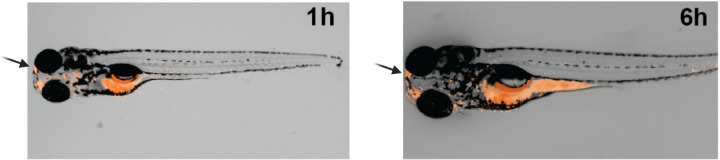
Time course of CX2 (8ARG-Cx43p, corresponding to the C-terminal domain sequence of Cx43) crossing the blood-brain barrier (BBB) efficiently in a 7-day-old zebrafish larva. The carboxytetramethylrhodamine (TAMRA)-labeled peptide showed fluorescent signal detection (indicated by red color) in the brain and throughout the body at 1 h and 6 h post-treatment, as observed by fluorescence microscopy.

To assess biodistribution, both the Tripeptide and CX2 were fluorescently labeled and tracked at four different time points: 1 h, 6 h, 18 h, and 24 h. CX2 exhibited strong fluorescence in the brain at 1 h post-treatment, persisting at 6 h (Fig 5). Additionally, CX2 fluorescence was detectable throughout the body, indicating successful systemic absorption by the zebrafish larvae. In contrast, the Tripeptide did not penetrate the brain, predominantly remaining localized in the zebrafish intestine (S1 Fig).

## Discussion

Despite the availability of pharmacological treatments, uncontrolled seizures remain a significant clinical concern underscoring the necessity for further research into novel strategies for seizure managing [3,5]. Seizures arise from complex processes involving multiple factors and affect various cellular pathways [29]. Given their critical roles in epilepsy pathogenesis and seizure responses, inflammation, and cell death pathways were specifically targeted in this study [30–33]. To this end, we evaluated two peptides, the Tripeptide (p-BTX-I) and CX2 (a Cx43-derived peptide), assessing their potential therapeutic effects on these pathaways [23,24,34].

Regarding the Tripeptide, we found that the 10 µg/mL concentration significantly increased swimming activity. Although not statistically significant, the number of seizures was also higher at the same concentration (Fig 1). Interestingly, from the molecular perspective, the lower doses, 10 µg/mL and 5 µg/mL, downregulated *il1b* and *casp9* genes suggesting beneficial effects on both inflammation and apoptotic pathways (Fig 3 A and C). The reduction in *il1b* levels indicates that the Tripeptide may have anti-inflammatory properties, potentially decreasing seizure-related neuroinflammation [32,35]. Additionally, decreased *casp9* implies reduced activation of the intrinsic apoptotic pathway, commonly triggered by mitochondrial dysfunction, oxidative stress, and excitotoxicity during seizures [30,33]. The significant effects observed at lower doses may indicate that the Tripeptide achieves its maximum neuroprotective potential at these concentrations. Indeed, the Tripeptide has previously been related to neuroprotection through modulation of neuroinflammatory pathways, protecting cells from neurotoxicity [23–24].

Although our findings suggest a neuroprotective response to seizure, these molecular effects were not clearly reflected in behavior outcomes or *c-fos* levels (Figs 1 and 3F). While the *c-fos* gene is a well-established marker of neuronal activity [36], its increased expression at the 10 µg/mL dose aligns with observed behavior but contrasts with the reduced expression of *il1b* and *casp-9* genes. This discrepancy warrants cautious interpretation since *c-fos* is also involved in multiple signaling pathways [36]. Additionally, the Tripeptide did not significantly affect *casp3a and baxa* levels compared to the PTZ group (Fig 3B and D). However, at 25 µg/mL, it reduced the *bcl2a* levels (Fig 3E), suggesting that higher concentrations may diminish its protective efficacy. This finding aligns with the observed reduction in *il1b* and *casp9* transcripts, further supporting a potential dose-dependent effect of the Tripeptide.

Concerning the Tripeptide’s biodistribution, it was primarily detected in the zebrafish larvae intestine (S1 Fig). This observation may result from the conjugated fluorophore (tetramethylrhodamine-TMR), potentially interfering with absorption or limiting its crossing of the blood-brain barrier to reach the CNS.

Regarding CX2, our results demonstrated that the treatment before PTZ-induced seizures downregulated the *il1b, cox1, tnfa, and il16* genes (Fig 4), indicating its significant role in inflammation regulation during seizure episodes [35]. Inflammatory responses involve various mediators, including interleukins (ILs), interferons (IFNs), tumor necrosis factors (TNFs), and growth factors. *IL1B*, *TNF*-α, and *IL6* genes are among the most studied inflammatory cytokines in the CNS [32]. Following seizure episodes, genes such as *IL1B*, *IL2*, *IL6*, *TNF*-α, and rapidly increase from low baseline levels, contributing to detrimental synaptic changes and neuronal hyperexcitability [35,37,38]. In this context, CX2 may attenuate these inflammatory responses, potentially reducing neuronal hyperexcitability and synaptic dysfunction associated with seizures. Ultimately, our findings suggest that CX2 could help mitigate the detrimental impact of seizures.

In contrast, the *cox2a* gene expression was upregulated compared to the PTZ group (Fig 4E). *COX2,* which is responsible for prostaglandin synthesis, typically increases during seizures [39–41]. Although COX2 inhibitors have been investigated as potential therapeutic agents, their efficacy remains controversial due to variability in inhibitor types and administration timing [40–42]. Studies in rats and zebrafish indicate that *cox1* may be more critical in seizure management [43–44]. For instance, Barbalho *et al*. demonstrated beneficial effects of *cox-1* inhibition on seizure control in zebrafish larvae, whereas *cox-2* inhibition showed no impact [43]. In our study, CX2 reduced *cox1* transcript levels (Fig 4D); however, despite this molecular finding, no clear behavioral impact was observed (Fig 2).

Although quantification of swimming activity showed no significant differences among groups, the frequency of seizures, indicated by loss of posture, tended to decrease at the 0.01 µM CX2 concentration, suggesting a potential biological effect despite lacking statistical significance (Fig 2A). This tendency was noted even though overall swimming activity (distance and velocity) remained comparable to the PTZ group (Fig 2), highlighting a direction for future investigation. While visual observation can be subjective, it still offer valuable insights [17,19].

CX2 exhibited rapid absorption and widespread biodistribution, detected in the brain as early as 1 hour post-administration (Fig 5), notably within the optic tectum, a region associated with increased neuronal activity during seizures [45]. Evaluating *c-fos* as a neuronal activation marker, we observed that the PTZ group and all three treatment groups showed significant differences compared to the control group, suggesting enhanced neuronal activation (Fig 4E). Although not statistically significant, the 0.1 µM CX2 concentration showed comparatively lower *c-fos* expression than the PTZ group (Fig 4E). Further research is needed to clarify the biological relevance of these observations.

The limitations of this study include the sample size and the reliance on visual scoring to assess seizure frequency. Additionally, seizure induction was restricted to 20 minutes, as described by Baraban et al., 2005 [19], limiting exploration of prolonged seizure exposure, broader peptide concentration ranges, and electrophysiological recordings to detect seizure-like discharges [18]. Furthermore, we evaluated only immediate effects post-seizure induction.

Despite these limitations, our findings highlight the potential of both peptides, especially CX2, in reducing post-seizure neuroinflammation. Future studies should investigate long-term effects on seizure control and epileptogenesis in zebrafish and mammalian models. Moreover, additional research should explore the role of these peptides in modulating neurotransmitter systems, such as glutamatergic and GABAergic activity, among others.

## Conclusion

Our study provides evidence that Tripeptide p-TBX-I and CX2 (a Cx43-derived peptide) can modulate gene expression and reduce epileptic seizure response in zebrafish. The Tripeptide decreased *casp9* and *il1b* gene expression, highlighting its role in regulating cellular stress response and neuroinflammation. Meanwhile, CX2 demonstrated significant anti-inflammatory properties by reducing key inflammatory gene expressions, which may mitigate seizure-induced neuroinflammation. Additionally, CX2 displayed extensive biodistribution within zebrafish brains and bodies. Collectively, these findings suggest both peptides are promising candidates for developing novel anti-seizure therapies. Further studies are necessary to assess their long-term efficacy, mechanisms of action, and potential translational applications.

## Supporting information

S1 Fig Time course of the Tripeptide in a 7-days post fertilization zebrafish larva. The tetramethylrhodamine-TMR labeled peptide was not detected in the brain through fluorescence microscopy.
(TIF)
