# Supplementary figures and images for: Effects of two different peptides on pentylenetetrazole-induced seizures in larval zebrafish
Source: PLoS One. 2025 Apr 25;20(4):e0308581. doi: 10.1371/journal.pone.0308581 (PMC12026968; doi:10.1371/journal.pone.0308581)

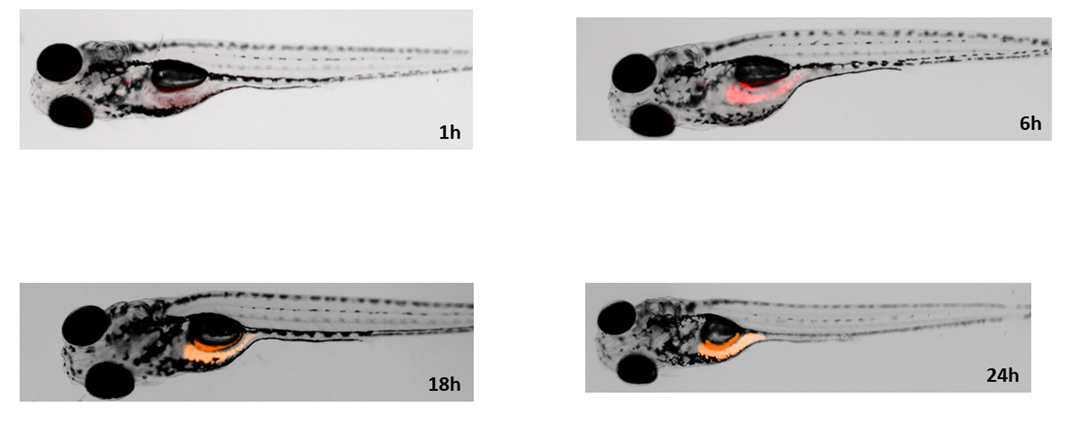

Supplement: S1 Fig — The tetramethylrhodamine-TMR labeled peptide was not detected in the brain through fluorescence microscopy. (TIF) [file pone.0308581.s001.tif]
